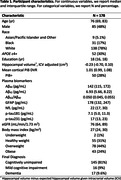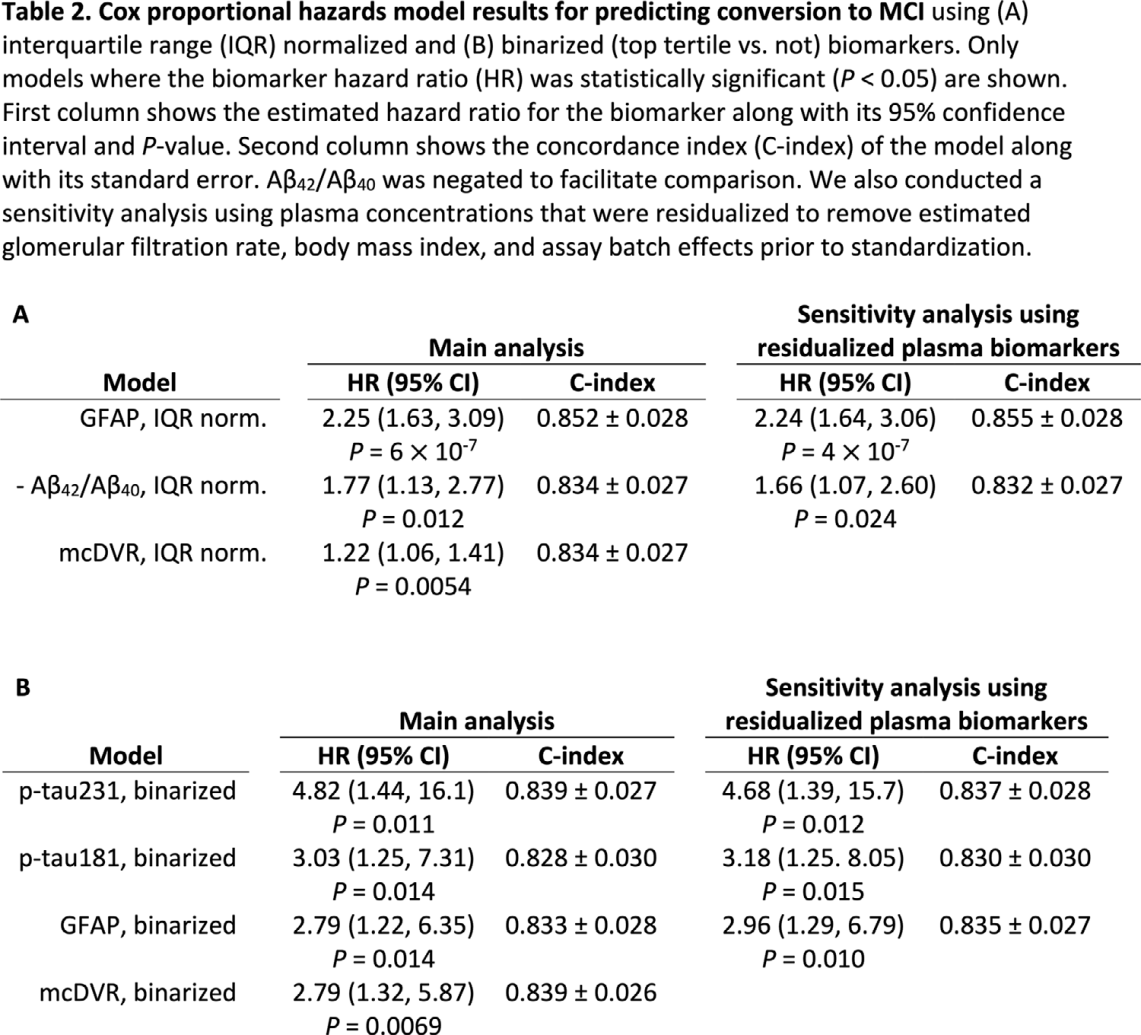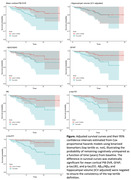# Comparison of amyloid positron emission tomography, hippocampal volume, and plasma biomarkers in predicting conversion to mild cognitive impairment

**DOI:** 10.1002/alz.093667

**Published:** 2025-01-09

**Authors:** Murat Bilgel, Yang An, Ishaan Shah, Keenan A. Walker, Abhay Moghekar, Nicholas J. Ashton, Przemyslaw Radoslaw Kac, Thomas K Karikari, Kaj Blennow, Henrik Zetterberg, Madhav Thambisetty, Susan M. Resnick

**Affiliations:** ^1^ National Institute on Aging, National Institutes of Health, Baltimore, MD USA; ^2^ National Institute on Aging, Baltimore, MD USA; ^3^ Laboratory of Behavioral Neuroscience, National Institute on Aging, Intramural Research Program, Baltimore, MD USA; ^4^ Johns Hopkins University School of Medicine, Baltimore, MD USA; ^5^ Centre for Age‐Related Medicine, Stavanger University Hospital, Stavanger Norway; ^6^ Department of Psychiatry and Neurochemistry, Institute of Neuroscience and Physiology, The Sahlgrenska Academy at the University of Gothenburg, Mölndal Sweden; ^7^ Department of Psychiatry, School of Medicine, University of Pittsburgh, Pittsburgh, PA USA; ^8^ Clinical Neurochemistry Laboratory Sahlgrenska University Hospital, Mölndal Sweden; ^9^ Hong Kong Center for Neurodegenerative Diseases, Hong Kong China

## Abstract

**Background:**

We examined whether brain amyloid PET, hippocampal volume, or plasma biomarkers are better predictors of conversion to mild cognitive impairment (MCI).

**Method:**

In the Baltimore Longitudinal Study of Aging (BLSA), plasma Aß42, Aß40, glial fibrillary acidic protein (GFAP), and neurofilament light chain (NfL) concentrations were measured using Quanterix Simoa Neurology 4‐plex E. Plasma p‐tau181 and p‐tau231 concentrations were measured using in‐house Simoa assays at University of Gothenburg. Brain amyloid was quantified using mean cortical 11C‐Pittsburgh compound B PET distribution volume ratio (mcDVR). Hippocampal volume was calculated from T1‐weighted MRI and adjusted for intracranial volume. Of 178 cognitively unimpaired (CU) participants with both plasma and neuroimaging measurements at baseline, 33 developed MCI during follow up (median time to MCI onset: 3.7 [interquartile range (IQR) 2.1‐6.2] years), 17 of whom had elevated brain PET amyloid at baseline. We used Cox proportional hazards models to examine the association between risk of conversion to MCI (time to conversion right‐censored at death or last visit for CU participants) and baseline plasma or neuroimaging biomarker, adjusting for age, sex, race, and education. To enable comparison of hazard ratios (HRs), we negated plasma Aß42/Aß40 and hippocampal volume, and standardized each biomarker using statistics calculated in a cross‐sectional dataset of CU individuals aged 60‐80 (preferring the visit closest to age 70 per participant) drawn from the larger BLSA. For standardization, we examined subtracting the median and dividing by the IQR and binarization (top tertile vs. not).

**Result:**

In analyses using IQR normalized scores, mcDVR and plasma Aß42/Aß40 and GFAP had statistically significant HRs (Table 2). In binarized biomarker analyses, mcDVR and plasma GFAP, p‐tau181, and p‐tau231 were statistically significant. Using all plasma biomarkers together yielded the highest concordance: 0.877±0.026 and 0.861±0.026 for the continuous and binarized biomarker analyses, respectively.

**Conclusion:**

Plasma biomarkers provide information beyond demographics regarding the prediction of MCI conversion within 4 years. Risk prediction conveyed by several individual plasma biomarkers exceeded that of brain amyloid PET. Plasma GFAP was consistently identified as contributing to the prediction of MCI conversion, with Aß42/Aß40 and p‐tau exhibiting statistically significant associations in a subset of analyses.